# A new perspective in predicting fall risk in non-parkinsonian geriatric patients: the magnetic resonance parkinsonism index

**DOI:** 10.1186/s12877-026-08112-3

**Published:** 2026-08-12

**Authors:** Ilknur Seker Kaya, Kubra Bahar Baykan, Yilmaz Onal, Bugra Umut Kaya, Adil Aytac, Suleyman Emre Kocyigit

**Affiliations:** 1 Department of Geriatric Medicine, Balikesir University Faculty of Medicine, Merkez, Paşaköy, Bigadiç yolu üzeri, Altıeylül, Balikesir, 10145 Türkiye; 2 Department of Radiology, Balikesir University Faculty of Medicine, Balikesir, Türkiye

**Keywords:** Geriatric syndromes, Recurrent falls, Magnetic resonance parkinsonism index

## Abstract

**Objectives:**

Falls are a major geriatric syndrome associated with significant morbidity and mortality in older adults. The Magnetic Resonance Parkinsonism Index (MRPI) is a neuroimaging biomarker reflecting brainstem structural alterations; however, its role in fall risk among non-parkinsonian older adults remains unclear. This study aimed to determine whether the MRPI is independently associated with recurrent falls in older adults.

**Methods:**

This retrospective cross-sectional study included 250 adults aged ≥ 65 years who underwent brain magnetic resonance imaging (MRI) and comprehensive geriatric assessment. Participants were categorised as fallers and non-fallers. MRPI values were calculated using segmentation-based measurements from three-dimensional T1-weighted MRI. Logistic regression models were used to evaluate the independent association between MRPI and recurrent falls after adjustment for potential confounders.

**Results:**

A total of 250 participants (171 women, 79 men) were included, of whom 103 (41.2%) had recurrent falls. MRPI values were higher in fallers than in non-fallers (13.22 vs. 12.74, *p* = 0.027). The MRPI was positively correlated with age, number of falls, number of medications, Timed Up and Go test time, and frailty, whereas it was negatively correlated with the Tinetti Performance-Oriented Mobility Assessment score, grip strength, and functional status (*p* < 0.05). The MRPI was independently associated with falls across all multivariable models, including the fully adjusted model (odds ratio, 1.61; 95% confidence interval, 1.02–2.48; *p* = 0.031).

**Conclusion:**

The MRPI is independently associated with falls in non-parkinsonian older adults and may represent a novel neuroimaging biomarker for fall risk prediction.

## Introduction

Falls are a multifactorial geriatric syndrome resulting from the interaction of intrinsic and extrinsic risk factors in the setting of age-related physiological decline, with incidence increasing markedly after the age of 65 years [1]. Falls and related injuries are common and potentially preventable causes of functional impairment, morbidity, and increased healthcare utilisation in older adults [2].

Recent clinical and epidemiological studies have demonstrated a strong association between gait performance and cognitive function in older adults. Alterations in gait characteristics have been associated with falls, as well as with the development of dementia and functional decline [3]. These findings suggest that gait and balance control are not solely dependent on the musculoskeletal system but are also closely related to the integrity of the central nervous system and its underlying neural networks [3]. Therefore, a better understanding of the relationship between early gait changes and cognitive decline may help identify older individuals at increased risk of falls and progressive neurological disorders [4].

Magnetic resonance imaging (MRI) is widely used as a neuroimaging modality to assist in the differentiation of atypical parkinsonian syndromes from Parkinson’s disease (PD), particularly playing a key role in distinguishing progressive supranuclear palsy (PSP) from PD [5, 6]. The magnetic resonance parkinsonism index (MRPI) is a neuroimaging biomarker based on morphometric measurements of brainstem structures, including the midbrain, pons, and cerebellar peduncles [7]. The MRPI reflects structural alterations in brainstem regions that play a critical role in gait regulation and postural control and may therefore provide insight into the mechanisms underlying balance impairment and fall risk. However, the potential relationship between the MRPI and falls in older adults without parkinsonism has not been sufficiently investigated. Considering that brain imaging is often performed as part of the routine clinical evaluation of older adults presenting with falls, assessing the MRPI from existing imaging data may offer additional information regarding the neuroanatomical mechanisms underlying fall risk and could serve as a potential marker to support clinical assessment.

This study investigated the association between the MRPI and fall risk in non-parkinsonian older adults and to determine whether the MRPI serves as an independent neuroimaging biomarker for falls beyond traditional clinical and functional risk factors.

## Materials and methods

### Study design

This retrospective cross-sectional study included 250 patients aged ≥ 65 years who attended our outpatient clinic between November 2022 and March 2026 and had available brain MRI scans. All participants underwent a comprehensive geriatric assessment. Owing to the retrospective design of the study, the requirement for informed consent was waived by the ethics committee. Brain MRI examinations had been performed before study enrolment as part of routine clinical evaluation for cognitive complaints, dizziness, gait disturbance, headache, suspected cerebrovascular disease, or other clinical indications.

### Inclusion criteria

The study included patients aged ≥ 65 years who visited our centre for any reason, had available brain MRI data, and did not meet any of the exclusion criteria.

### Exclusion criteria

Patients younger than 65 years and those who declined participation were excluded. To avoid potential confounding of MRPI measurements, patients with parkinsonian disorders (PD, PSP, or MSA) were not included. Additional exclusion criteria were severe musculoskeletal or neuromuscular disorders limiting mobility, falls secondary to cerebrovascular events, epilepsy or trauma, acute systemic illness, acute cardiovascular or cerebrovascular events, active malignancy, severe anaemia, decompensated liver cirrhosis, and inadequate MRI quality for reliable MRPI measurement.

### Patient characteristics

Demographic data, including age, sex, level of education, marital status, living arrangement, and body mass index (BMI), were recorded for each participant. Comorbidities, including hypertension, diabetes mellitus, chronic heart disease, osteoarthritis, osteoporosis, depression, dementia, and chronic lung disease, were also documented. All medications were systematically reviewed and categorised.

Laboratory parameters, including glucose, haemoglobin, estimated glomerular filtration rate (eGFR), sodium, potassium, vitamin B12, 25-hydroxyvitamin D, ferritin, and thyroid-stimulating hormone (TSH), were retrospectively evaluated. Anemia was defined according to the World Health Organization criteria as a haemoglobin concentration < 13.0 g/dL in men and < 12.0 g/dL in women.

Geriatric syndromes, including probable sarcopenia, recurrent falls (> 2 falls in the last year), malnutrition, polypharmacy, geriatric depression, dementia, and frailty, were assessed by an experienced geriatrician. The diagnoses of dementia and geriatric depression were established according to the Diagnostic and Statistical Manual of Mental Disorders, Fifth Edition (DSM-5) criteria [8].

### Comprehensive geriatric assessment parameters

All patients included in the study underwent a comprehensive geriatric assessment. Mood was assessed using the Yesavage Geriatric Depression Scale (YGDS) [9]. Functional status was evaluated using the Lawton–Brody Instrumental Activities of Daily Living (IADLs) [10] and the Barthel Activities of Daily Living (BADLs) [11]. Balance and gait were assessed using the Tinetti Performance-Oriented Mobility Assessment (POMA) [12]. Frailty was assessed using the Fried frailty phenotype criteria [13]. Sarcopenia was assessed according to the European Working Group on Sarcopenia in Older People 2 (EWGSOP2) criteria, and muscle strength was evaluated using handgrip dynamometry [14]. Nutritional status was evaluated using the Mini Nutritional Assessment–Short Form (MNA-SF) [15] and cognitive function was assessed using the standardised Mini-Mental State Examination (sMMSE) [16].

### MRPI measurement

Brain MRI was requested solely according to the treating physician’s clinical judgment and was not performed systematically for all outpatients. MRPI values were derived using a segmentation-based measurement approach on high-resolution three-dimensional T1-weighted magnetic resonance images. All image processing and measurements were performed using three-dimensional (3D) Slicer (version 5.6.1; www.slicer.org), an open-source medical image analysis platform. All 3D T1-weighted images were visually inspected for motion artefacts and anatomical distortions prior to analysis. Image volumes were analysed in native space without spatial normalisation to preserve individual anatomical proportions.

**Fig. 1:**
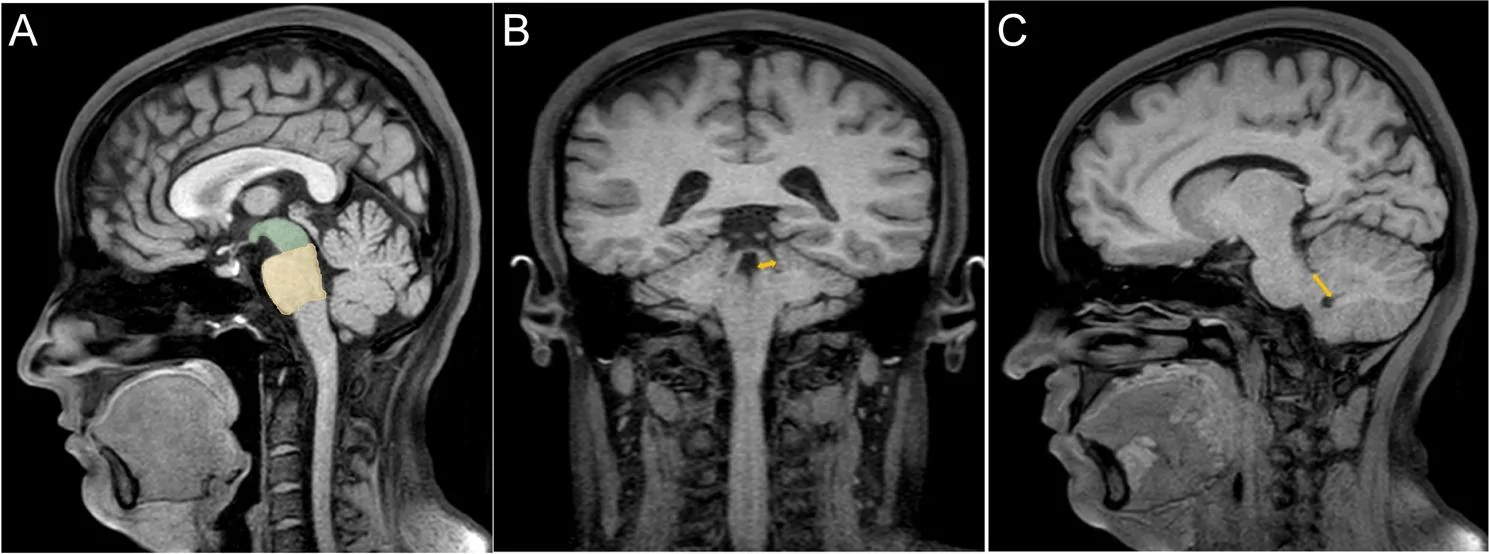
Measurement of MR Parkinsonism Index (MRPI) parameters on structural MRI. **A** Mid-sagittal T1-weighted image demonstrating manual segmentation of the midbrain area (green) and pons area (yellow), used to calculate the pons-to-midbrain area ratio. **B** Coronal T1-weighted image illustrating measurement of the left superior cerebellar peduncle (SCP) width (double-headed yellow arrow). **C** Sagittal T1-weighted image showing measurement of the middle cerebellar peduncle (MCP) width (double-headed yellow arrow).

Segmentation and measurements of the midbrain, pons, superior cerebellar peduncles (SCP), and middle cerebellar peduncles (MCP) were performed using the Segment Editor module. Anatomical delineation was conducted with multiplanar (axial, sagittal, and coronal) guidance. Manual segmentation was used as the primary method, with optional refinement using thresholding, island removal, and smoothing tools when required. All segmentations were reviewed on a slice-by-slice basis to ensure anatomical accuracy. Midbrain and pons areas were measured on the midsagittal plane by extracting the cross-sectional areas of each structure. SCP width was measured bilaterally on coronal images at the level of maximal peduncular thickness, perpendicular to the long axis of the peduncle, and the mean value was used for analysis. MCP width was measured bilaterally on axial images at the level of the pontine tegmentum, and the mean value was recorded (Fig. 1).

The MRPI was calculated using the following formula:

$$\begin{aligned}\mathrm{MRPI}&=\left(\text{pons area}/\text{midbrain area}\right)\\&\times\left(\text{MCP width}/\text{SCP width}\right)\end{aligned}$$

The MRPI was calculated as the ratio of the pons-to-midbrain area multiplied by the ratio of the MCP-to-SCP width. All measurements were performed by an experienced radiologist blinded to the clinical data.

### Statistical analysis

The participants were divided into two groups according to fall status (fallers and non-fallers). Categorical variables are presented as percentages (%), and continuous variables are expressed as mean ± standard deviation. The distribution of continuous variables was assessed using the Kolmogorov–Smirnov test. Because the variables were not normally distributed, non-parametric tests were applied. Comparisons between groups were performed using the chi-square test for categorical variables and the Mann–Whitney U test for continuous variables. Spearman correlation analysis was performed to evaluate the relationships between the MRPI and demographic, clinical, and laboratory variables. The association between the MRPI and falls was evaluated using logistic regression analysis. Prior to multivariable logistic regression analyses, model assumptions were evaluated. Multicollinearity among independent variables was assessed using variance inflation factors (VIFs) and tolerance values. Model calibration was evaluated using the Hosmer–Lemeshow goodness-of-fit test. Influential observations and potential outliers were examined using standardized residuals and Cook’s distance. No substantial violations of model assumptions were identified; therefore, no remedial measures were required. Odds ratios (ORs) with 95% confidence intervals (CIs) were calculated across sequential models: Model 0 (unadjusted); Model 1 (adjusted for demographic variables, including age and sex); Model 2 (adjusted for Model 1 plus comorbidities, including chronic heart disease, chronic lung disease, and osteoporosis); Model 3 (adjusted for Model 2 plus medication use, including antidepressants, insulin, and antimuscarinic drugs); and Model 4 (adjusted for Model 3 plus functional and geriatric parameters, including the Timed Up and Go test (TUG) and Fried frailty score). Statistical significance was set at a p-value of < 0.05. All statistical analyses were conducted using IBM SPSS Statistics version 22.0 (IBM Corp., Armonk, NY, USA).

## Results

**Fig. 2:**
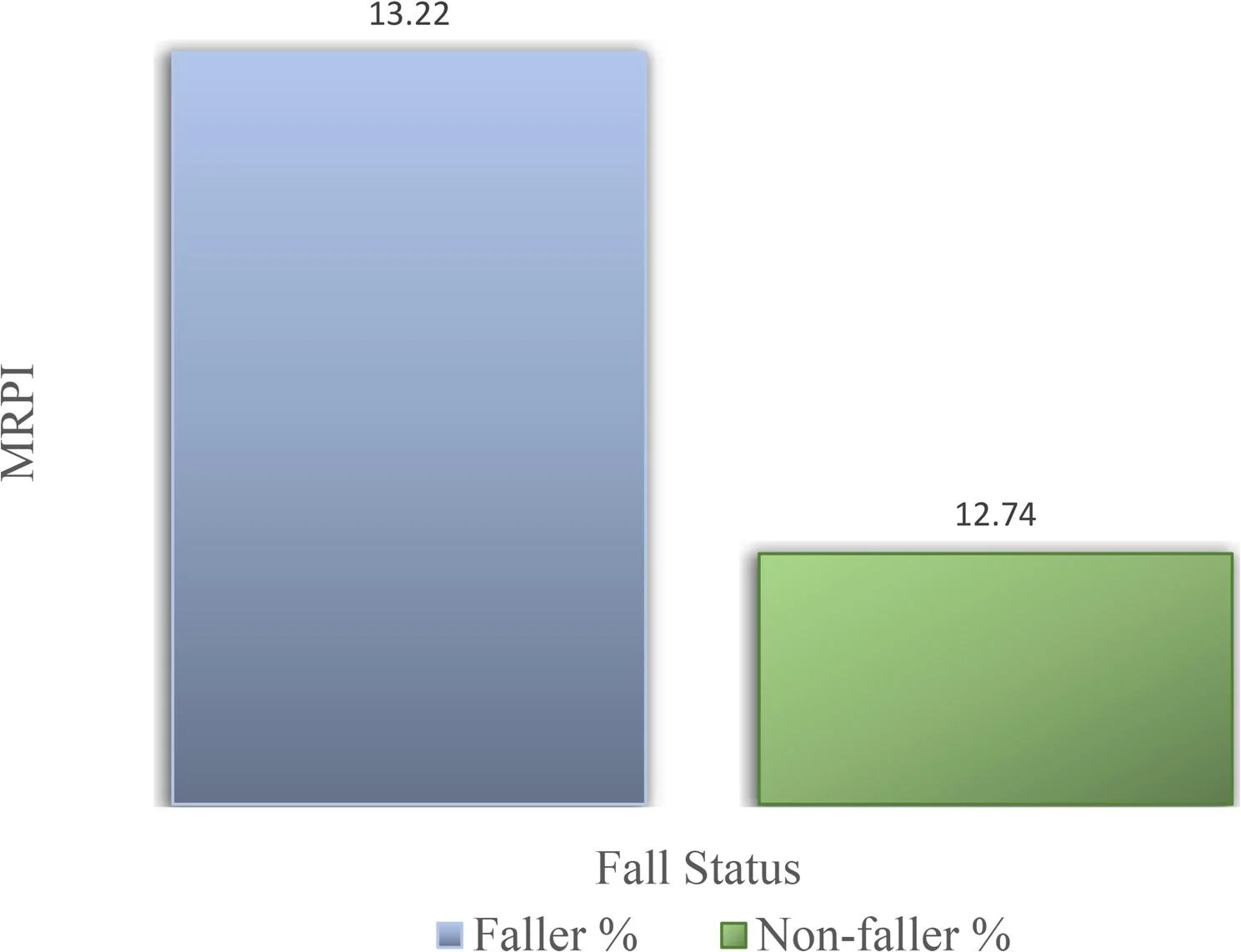
Comparison of Magnetic Resonance Parkinsonism Index (MRPI) values according to fall status (*p*= 0.027)

In total, 250 geriatric patients (171 women and 79 men) were included in the study; 103 (41.2%) had a history of falls, while 147 (58.8%) did not. MRPI values were higher in participants with a history of falls than in those without (13.22 vs. 12.74, *p* = 0.027) (Fig. 2). No statistically significant differences were observed between fallers (*n* = 103) and non-fallers (*n* = 147) with respect to age, sex, education level, marital status, living arrangement, and BMI (all *p* > 0.05) (Table 1). Fallers had a significantly higher prevalence of chronic heart disease, osteoporosis, and chronic lung disease than did non-fallers (*p* = 0.005, *p* = 0.027, and *p* = 0.009, respectively). No statistically significant differences were observed between the groups in terms of hypertension, diabetes mellitus, osteoarthritis, depression, and dementia (all *p* > 0.05) (Table 1). Fallers also had higher use of antidepressants, sodium-glucose cotransporter 2 (SGLT2) inhibitors, insulin, and antimuscarinic agents than did non-fallers (*p* = 0.007, *p* = 0.044, *p* = 0.008, and *p* = 0.026, respectively). No statistically significant differences were observed between the groups in the use of renin–angiotensin–aldosterone system blockers (RAAS), diuretics, calcium channel blockers, beta-blockers, alpha-blockers, antipsychotics, or antihistamines (all *p* > 0.05) (Table 1). Haemoglobin levels and the frequency of anemia were different in fallers than in non-fallers (*p* = 0.010 and *p* = 0.002, respectively). Number of drugs, POMA score, FRIED frailty scale, TUG duration, BADLs and IADLs score were statistically different between two groups (all *p* < 0.05). No statistically significant differences were observed between the groups with respect to glucose, eGFR, sodium, potassium, vitamin B12, 25-hydroxyvitamin D, ferritin, or TSH levels (all *p* > 0.05) (Table 1). Spearman correlation analysis demonstrated that the MRPI was positively correlated with age (ρ = 0.850, *p* < 0.001), number of falls (ρ = 0.133, *p* = 0.036), number of medications (ρ = 0.146, *p* = 0.021), TUG duration (ρ = 0.336, *p* < 0.001), and Fried frailty score (ρ = 0.240, *p* < 0.001).

**Table 1:** Comparison of demographic characteristics, comorbid diseases, and laboratory findings according to Magnetic Resonance Parkinsonism Index (MRPI)

	Faller *n* = 103	Non-faller *n* = 147	*p* value
Demographic Features			
Age [mean (min-max)]	76.87 (65–91)	75.16 (65–90)	0.920
Education year [median (IQR)]	5 (1)	5 (0)	0.291
Marital Status [Married, %]	53.7	51.5	0.337
Gender [female, %]	68	68.7	0.901
Living alone [%]	14.6	22.4	0.243
BMI (kg/m^2^) [mean (min-max)]	27.94 (17.9–46.0)	27.10 (15.5–43.8)	0.150
Comorbidities [%]			
Hypertension	74.8	64.6	0.089
Diabetes Mellitus	45.6	35.4	0.103
Chronic Heart Disease	42.7	25.9	**0.005**
Osteoarthritis	27.2	21.8	0.324
Osteoporosis	32.0	19.7	**0.027**
Depression	48.8	45.6	0.400
Dementia	42.2	41.8	0.953
Chronic Lung Disease	21.4	9.5	**0.009**
Drugs [%]			
Number of drugs [mean (min-max)	7.96 (0–18)	5.54 (0–22)	**< 0.001**
RAAS Blockers	54.4	45.6	0.171
Diuretics	40.8	38.8	0.750
Calcium Channel Blockers	25.2	25.2	0.990
Beta Blockers	41.7	31.3	0.089
Alfa- blockers	19.4	17	0.625
Antidepressants	48.5	31.5	**0.007**
Antipsychotic Drugs	15.5	12.2	0.455
SGLT-2 Inhibitors	12.6	5.4	**0.044**
Insulins	17.5	6.8	**0.008**
Antimuscarinic Drugs	16.5	7.5	**0.026**
Antihistamines	3.9	3.4	0.840
Laboratory findings [mean (min-max)]			
Hemoglobin [g/dL] Anemia (%)	12.43 (9.5–15.4) 47.5	12.89 (8.6–15.6) 28.5	**0.010** **0.002**
Glucose [mg/dL]	117.55 (70–432)	127.44 (66–372)	0.775
eGFR [mL/min]	63.46 (30.4–92.0)	68.31 (30.2–102.0)	0.140
Sodium [mmol/L]	138.42 (134–148)	138.98 (129–145)	0.072
Potassium (mmol/L)	4.34 (3.2–5.5)	4.38 (3.5–5.7)	0.974
Vitamin B12 [ng/L]	388.67 (70-2000)	474.88 (52-2000)	0.666
25-hydroxy Vitamin D [µg/L]	20.67 (4.3–53.7)	20.56 (4.2–63.6)	0.586
Ferritin [ng/mL]	59.95 (4.7-535.5)	80.61 (5.4-639.6)	0.525
TSH [mU/L]	1.52 (0.03–4.80)	1.59 (0.27–10.2)	0.873
Other Variables [mean (min-max)]			
Number of falls	4.12 (2–20)	0.24 (0–1)	**< 0.001**
MNA-SF score	11.23 (5–14)	11.64 (5–14)	0.235
POMA score	19.00 (2–28)	23.23 (7–28)	**< 0.001**
TUG duration (sec)	34.37 (9.1-185.2)	19.78 (8.7–59.8)	**< 0.001**
FRIED Frailty Scale	2.91 (0–5)	2.32 (0–5)	**< 0.001**
Grip Strength (kg)	16.50 (4–36)	16.82 (2–36)	0.125
BADLs	77.30 (30–100)	89.30 (50–100)	**< 0.001**
IADLs	13.51 (2–23)	15.42 (4–23)	**0.002**
sMMSE	20.51 (5–29)	20.27 (4–30)	0.875
Yesevage GDS	5.97 (0–15)	5.20 (0–14)	0.117

*BADLs* Basic Activities of Daily Living, *BMI* Body Mass Index, *eGFR* Estimated Glomerular Filtration Rate, *GDS* Geriatric Depression Scale, *IADLs* Instrumental Activities of Daily Living, *IQR* interquartile range, *MNA-SF* Mini-nutritional Assessment-Short Form, *POMA* Performance-oriented Mobilitiy Assessment, *RAAS* Renin-Angiotensin-Aldosterone System, *SD* Standard Deviation, *SGLT-2* Sodyum-Glucose Co-Transporter, *sMMSE* Standardized Mini-mental State Examination, *TSH* Thyroid-Stimulating Hormone, *TUG* Timed Up and Go Test. Bold values indicate statistical significance (p < 0.05).

By contrast, the MRPI showed negative correlations with haemoglobin (ρ = −0.167, *p* = 0.008), eGFR (ρ = −0.352, *p* < 0.001), POMA score (ρ = −0.319, *p* < 0.001), grip strength (ρ = −0.177, *p* = 0.006), BADLs score (ρ = −0.180, *p* = 0.004), and IADLs score (ρ = −0.186, *p* = 0.003). No statistically significant correlations were observed between the MRPI and MNA-SF, sMMSE, YGDS, or BMI (all *p* > 0.05) (Table 2). Although several correlations reached statistical significance, with the exception of age (ρ = 0.850), the observed correlation coefficients were generally negligible to small in magnitude (|ρ| < 0.40), indicating modest effect sizes. Therefore, these findings should be interpreted as reflecting weak bivariate associations rather than strong clinical relationships.

**Table 2:** Spearman correlation coefficients between Magnetic Resonance Parkinsonism Index (MRPI) with demographic, clinical, and laboratory variables

	ρ (rho) value CI 95 (%) [(min)-(max)]	*p* value
Age	0.850 [(0.812)-(0.881)]	< 0.001
Number of falls	0.133 [(0.009)-(0.253)]	**0.036**
Number of drugs	0.146 [(0.022)-(0.265)]	**0.021**
Hemoglobin	-0.167 [(-0.285)-(-0.044)]	**0.008**
Glucose [mg/dL]	-0.058 [(-0.181)-(0.067)]	0.365
eGFR [mL/min]	-0.352 [(-0.456)-(-0.238)]	**< 0.001**
Sodium [mmol/L]	-0.042 [(-0.165)-(0.082)]	0.508
Potassium (mmol/L)	-0.068 [(-0.190)-(0.057)]	0.284
Vitamin B12 [ng/L]	-0.084 [(-0.206)-(0.040)]	0.185
25-hydroxy Vitamin D [µg/L]	0.012 [(-0.112)-(0.136)]	0.851
Ferritin [ng/mL]	0.087 [(-0.037)-(0.209)]	0.176
MNA-SF score	-0.059 [(-0.182)-(0.066)]	0.352
POMA score	-0.319 [(-0.426)-(-0.203)	0.352
Grip strength	-0.177 [(-0.295)-(-0.054)]	**0.006**
TUG duration [sec]	0.336 [(0.221)-(0.442)]	**0.006**
Fried frailty score	0.240 [(0.119)-(0.354)]	**<0.001**
BADLs	-0.180 [(-0.297)-(-0.057)]	**<0.001**
IADLs	-0.186 [(-0.303)-(-0.063)]	**0.003**
sMMSE	-0.066 [(-0.189)-(0.059)]	**0.003**
Geriatric Depression Scale	0.025 [(-0.099)-(0.149)]	0.728
BMI [kg/m^2^ ± SD]	-0.059 [(-0.182)-(0.066)]	0.462

*BADLs* Basic Activities of Daily Living, *BMI* Body Mass Index, *eGFR* Estimated Glomerular Filtration Rate, *IADLs* Instrumental Activities of Daily Living, *MNA-SF* Mini Nutritional Assessment–Short Form, *POMA*: Performance-Oriented Mobility Assessment, *SD* Standard Deviation, *sMMSE* Standardized Mini-mental State Examination, *TUG* Timed Up and Go

Multivariable logistic regression analysis demonstrated that the MRPI was statistically associated with falls across all models. In the unadjusted model (Model 0), the MRPI was associated with an increased likelihood of falls (OR, 1.29; 95% CI, 1.04–1.61; *p* = 0.020). This association remained significant after adjustment for demographic variables in Model 1 (OR, 1.53; 95% CI, 1.07–2.18; *p* = 0.018) and persisted after further adjustment for comorbidities in Model 2 (OR, 1.48; 95% CI, 1.02–2.13; *p* = 0.035). In Model 3, which included medication use, the MRPI continued to show a statistically significant association with falls (OR, 1.55; 95% CI, 1.05–2.27; *p* = 0.024). This relationship remained robust in the fully adjusted model (Model 4), even after controlling for TUG duration and FRIED frailty scale (OR, 1.61; 95% CI, 1.02–2.48; *p* = 0.031). These findings indicate that the MRPI is independently associated with fall risk beyond conventional demographic, clinical, and functional factors (Tables 3 and 4).

**Table 3:** Logistic regression models examining the association between Magnetic Resonance Parkinsonism Index (MRPI) and falls

	Variables	βOdds ratio (OR)	95% Confidence Interval (CI)	*p* value
Model 0	MRPI	0.26 1.29	1.04–1.61	0.020
Model 1	MRPI	0.42 1.53	1.07–2.18	**0.018**
	Age	-0.03 0.97	0.92–1.02	0.241
	Sex	0.06 1.06	0.61–1.84	0.829
Model 2	MRPI	0.39 1.48	1.02–2.13	**0.035**
	Age	-0.04 0.96	0.91–1.01	0.143
	Sex	0.19 1.22	0.67–2.21	0.514
	Chronic Heart Disease	0.61 1.85	1.05–3.27	**0.033**
	Chronic Lung Disease	0.76 2.15	1.00-4.59	**0.048**
	Osteoporosis	0.69 1.99	1.05–3.77	**0.033**
Model 3	MRPI	0.43 1.55	1.05–2.27	**0.024**
	Age	-0.04 0.95	0.90-1.00	0.091
	Sex	0.44 1.55	0.83–2.92	0.167
	Chronic Heart Disease	0.63 1.88	1.03–3.42	**0.039**
	Chronic Lung Disease	0.75 2.12	0.95–4.73	0.064
	Osteoporosis	0.74 2.10	1.08–4.11	**0.029**
	Antidepressant drugs	0.72 2.06	1.16–3.66	**0.013**
	Insulin	1.36 3.89	1.60–9.49	**0.003**
	Antimuscarinik drugs	0.71 2.03	0.84–4.89	0.112
Model 4	MRPI	0.38 1.61	1.02–2.48	**0.031**
	Age	-0.06 0.94	0.88-1.00	0.054
	Sex	0.66 1.95	0.98–3.87	0.056
	Chronic Heart Disease	0.41 1.50	0.80–2.83	0.203
	Chronic Lung Disease	0.82 2.27	1.00-5.14	**0.047**
	Osteoporosis	0.70 2.01	0.98–4.12	0.055
	Antidepressant drugs	0.78 2.19	1.19–4.01	**0.011**
	Insulin	1.06 2.89	1.15–7.26	**0.024**
	Antimuscarinik drugs	0.52 1.68	0.66–4.29	0.273
	TUG test	0.02 1.02	1.00-1.04	**0.018**
	FRIED frailty scale	0.11 1.11	0.88–1.41	0.346

Model 0: Unadjusted

Model 1: Adjusted for demographic variables including age and sex

Model 2: Adjusted for model 1 plus the presence of chronic heart disease, chronic lung disease, and osteoporosis

Model 3: Adjusted for Model 2 plus the use of antidepressants, insulin, and antimuscarinic drugs

Model 4: Adjusted for Model 3 plus Timed Up and Go test and Fried frailty score

**Table 4:** Performance measures of the sequential multivariable logistic regression models

Model	X_2_	over all *p*	Nagelkerke *R*^2^	Hosmer-Lemeshow *p*	AUC
0	5.66	0.018	0.03	0.972	0.58
1	7.15	0.014	0.04	0.954	0.64
2	22.08	0.001	0.11	0.767	0.69
3	40.89	< 0.001	0.20	0.725	0.72
4	55.18	< 0.001	0.27	0.714	0.79

## Discussion

The present study demonstrated a significant association between the MRPI and recurrent falls in older adults. Higher MRPI values were associated with an increased likelihood of recurrent falls and this relationship remained significant after adjustment for confounders. In addition, the MRPI was correlated with several clinically relevant indicators of fall risk, including impaired balance (POMA), prolonged mobility time (TUG), reduced muscle strength, and frailty status. These findings suggest that MRPI may reflect structural alterations in brainstem regions involved in gait and postural control. Although MRPI has been extensively studied in parkinsonian disorders, its association with falls in non-parkinsonian older adults has not previously been investigated. Our findings therefore provide novel evidence supporting MRPI as a potential neuroimaging marker of fall risk in older adults.

Falls are multifactorial events resulting from the interaction of intrinsic and extrinsic risk factors, with age-related declines in sensory, cognitive, and motor function contributing to impaired compensatory responses [17]. As one of the leading causes of morbidity and mortality in older adults, falls increase in frequency with advancing age [18]. Given the strong correlation between the MRPI and age observed in a previous study, this finding suggests that the MRPI may capture age-related structural changes more sensitively than chronological age alone, potentially explaining the lack of a direct association between age and falls. Although previous studies have consistently identified advanced age, dementia, and depression as important risk factors for falls [19], these variables were not associated with recurrent falls in our cohort. This discrepancy may be explained by the relatively homogeneous nature of our study population, which consisted exclusively of geriatric outpatients with a high baseline prevalence of geriatric syndromes. Consequently, the limited variability in these characteristics may have reduced the ability to detect between-group differences. Furthermore, MRPI showed a strong correlation with age while remaining independently associated with falls, suggesting that it may reflect age-related structural brainstem changes more sensitively than chronological age alone.

Multimorbidity is highly prevalent in older adults, and a growing body of evidence has demonstrated a positive association between the accumulation of chronic conditions and an increased risk of falls in this population [20]. Osteoporosis is associated with reduced muscle strength, postural alterations, and impaired balance, all of which may collectively increase the risk of falls. Fragility fractures typically occur following low-energy falls and are subsequently associated with increased disability, morbidity, and mortality [21]. In a previous study, chronic heart disease, osteoporosis, and chronic lung disease were significantly more prevalent among fallers than among non-fallers. The observed associations may be attributed to the combined impact of these conditions on haemodynamic regulation, muscle strength, and functional capacity. Collectively, these impairments may disrupt balance, limit mobility, and compromise postural control, ultimately increasing fall susceptibility in older adults.

In addition, the MRPI showed weak but statistically significant positive correlations with the number of falls and the number of medications, suggesting a potential link with fall risk and polypharmacy. Importantly, although several correlations reached statistical significance, their effect sizes were generally negligible to small. Therefore, these associations should not be overinterpreted as indicating strong clinical relationships at the individual level. Rather, they suggest that MRPI is associated with multiple domains of geriatric health, each contributing only modestly in isolation. The persistence of the association between MRPI and recurrent falls in multivariable logistic regression analyses suggests that its clinical relevance is better reflected by its independent predictive value than by the magnitude of individual bivariate correlations. The concurrent use of multiple medications, commonly referred to as polypharmacy, is highly prevalent among older adults with multimorbidity [22]. Polypharmacy is a well-recognised contributor to fall risk in older adults, primarily through its impact on balance, cognition, and haemodynamic stability. The use of multiple medications increases the likelihood of adverse effects such as dizziness, sedation, and orthostatic hypotension, while also reflecting underlying multimorbidity and frailty. Furthermore, a substantial body of evidence has evaluated fall risk according to specific medication classes, consistently demonstrating that certain drug groups—particularly psychotropic, antihypertensive, and anticholinergic agents—are associated with an increased risk of falls [23, 24]. In a previous study, the use of antidepressants, SGLT2 inhibitors, insulin, and antimuscarinic agents was associated with falls. Antidepressants and antimuscarinic agents may increase fall risk through sedative and anticholinergic effects, as well as cognitive impairment, whereas insulin and SGLT2 inhibitors may contribute through hypoglycaemia, volume depletion, and haemodynamic instability. Together, these mechanisms may compromise balance and postural control, ultimately increasing fall susceptibility in older adults. In a previous study, the MRPI was found to predict falls independently of medications known to contribute to fall risk.

Frailty represents a multidimensional syndrome characterised by reduced physiological reserve and increased vulnerability to external stressors, and it is strongly associated with fall risk in older adults. This relationship may be mediated by a combination of factors, including probable sarcopenia, impaired balance, reduced gait speed, and diminished neuromuscular coordination. Furthermore, frailty is frequently accompanied by cognitive decline and impaired executive function, which may adversely affect the ability to adapt to environmental challenges. Together, these deficits contribute to impaired postural stability and an increased likelihood of falls [18, 25]. This finding is consistent with the positive correlation observed between the MRPI and frailty in a previous study and further supports the role of the MRPI as a marker of functional vulnerability.

In parkinsonism, freezing of gait—defined as a transient inability to initiate or maintain walking—together with balance impairment, reduced mobility, and lower extremity muscle weakness, significantly contributes to fall risk. In addition, fear of falling and cognitive impairment further exacerbate instability [26]. Consequently, approximately 60% of individuals with parkinsonism experience at least one fall annually, and nearly two-thirds of these individuals experience recurrent falls [27]. Differentiating PD from other neurodegenerative disorders, particularly PSP, remains challenging in the early stages because of subtle clinical features and variable responses to dopaminergic therapy; however, accurate diagnosis is essential for guiding treatment decisions and predicting prognosis [28]. Several quantitative MRI-derived metrics have been proposed as useful biomarkers for distinguishing PD from PSP [29]. Among these, the MRPI is widely recognised as one of the most reliable neuroimaging markers for supporting the clinical diagnosis of PSP [30, 31]. The MRPI has been shown to aid in the differential diagnosis of PSP from PD and the parkinsonian variant of MSA [32].

Importantly, patients with parkinsonian disorders were excluded from the present study. Therefore, our findings extend the potential clinical relevance of the MRPI beyond its established role in parkinsonian syndromes, suggesting that the MRPI may also capture subclinical brainstem alterations associated with fall risk in non-parkinsonian older adults. However, the ability of the MRPI to predict falls in individuals without parkinsonism indicates that this index may not be specific to PD and may instead be influenced by age-related structural changes—particularly atrophy of the midbrain and cerebellar peduncles, which are critical for motor control and postural regulation [27]. Given that MRPI is derived from morphometric measurements of these structures, increased MRPI values may reflect age-related brainstem atrophy and the resulting impairments in gait and balance. In line with this, Spearman correlation analysis demonstrated a strong positive association between the MRPI and age, supporting the notion that the MRPI may reflect age-related structural changes in the brainstem. These findings further suggest that the MRPI may represent a marker of biological ageing rather than chronological age alone. Accordingly, rather than being interpreted solely as a PD-specific assessment tool, the MRPI may provide a novel perspective in the evaluation of fall risk.

In a previous study, the MRPI demonstrated the associations with multiple functional parameters related to fall risk. Higher MRPI values were associated with poorer balance performance (lower POMA scores), reduced muscle strength, prolonged mobility time (TUG), and decreased functional independence, as reflected by lower ADL and IADL scores. In addition, the MRPI showed a positive correlation with frailty status. These findings suggest that MRPI may not only reflect structural brainstem alterations but also their functional consequences, particularly in terms of impaired balance, reduced mobility, and increased vulnerability. Collectively, these impairments represent key components of fall risk in older adults and may explain the observed association between the MRPI and falls.

Importantly, although the MRPI was strongly associated with age, it remained independently associated with falls after adjustment for age and other confounding variables; this finding suggests that the MRPI may be linked to fall risk through both age-related and age-independent mechanisms.

To our knowledge, this is the first study to investigate the association between MRPI and falls in non-parkinsonian older adults. MRPI remained significantly associated with falls across all regression models, including the fully adjusted model, indicating an association independent of demographic characteristics, comorbidities, medication use, and functional parameters. These findings suggest that MRPI may capture neuroanatomical alterations related to fall risk that are not fully reflected by conventional geriatric assessments and support its potential as a neuroimaging biomarker of fall susceptibility.

Furthermore, brain MRI is frequently performed as part of the routine aetiological evaluation in older adults presenting with falls. The ability to derive the MRPI from existing imaging data without additional image acquisition, radiation exposure, or substantial cost enhances its value as a practical and cost-effective adjunctive tool in clinical settings. Importantly, our findings should not be interpreted as supporting routine brain MRI solely for fall risk assessment in all older adults. Rather, MRPI may provide incremental prognostic information when brain MRI has already been obtained for appropriate clinical indications, such as cognitive impairment, gait disturbance, dizziness, or other neurological symptoms. In this context, MRPI represents a feasible neuroimaging biomarker that can be incorporated into the interpretation of existing MRI studies without increasing healthcare expenditures. To our knowledge, this is the first study to directly investigate the relationship between MRPI and fall risk, and our findings provide a novel contribution to the literature by addressing an important gap in current knowledge. From a clinical perspective, MRPI is not intended as a stand-alone diagnostic tool but rather as an adjunctive biomarker to complement comprehensive geriatric assessment. Identification of older adults with elevated MRPI values may support earlier implementation of individualized fall prevention strategies, including targeted balance and gait rehabilitation, strength training, medication review, environmental modifications, and closer clinical follow-up. Whether MRPI-guided risk stratification improves clinical outcomes or informs tailored intervention strategies remains to be established in prospective longitudinal studies and represents an important area for future research.

### Strength and limitations

A major strength of our study is that it is, to our knowledge, the first to demonstrate that the MRPI can predict falls in non-parkinsonian individuals, and that this association remains independent of potential confounding factors. However, this study has several limitations. First, because of its cross-sectional design, causal relationships between the MRPI and falls cannot be established. Second, the single-centre nature of the study may limit the generalisability of the findings. Third, only patients with available brain MRI were included, which may have introduced selection bias. Brain MRI had been obtained as part of routine clinical care rather than for research purposes, and patients without MRI may have represented individuals with milder clinical presentations or lower suspicion of neurological disease. Consequently, our cohort may not be fully representative of the general older adult population, and the findings should primarily be generalized to older adults undergoing clinical brain MRI evaluation. Another limitation is that multiple inferential statistical tests were performed without formal adjustment for multiple comparisons. This may have increased the probability of type I error, particularly for secondary and exploratory analyses. Therefore, these findings should be interpreted cautiously and considered hypothesis-generating until confirmed in larger independent studies.

## Conclusion

The MRPI may serve as a practical and cost-effective neuroimaging marker for recurrent fall risk, particularly in older adults with recurrent falls or pre-existing brain MRI—especially those with dementia—supporting its use in routine geriatric assessment.

## Data Availability

The datasets used and/or analysed during the current study are available from the corresponding author on reasonable request.

## Abbreviations

ADL: Activities of Daily Living
BADL: Basic Activities of Daily Living
BMI: Body Mass Index
CI: Confidence Interval
DSM-5: Diagnostic and Statistical Manual of Mental Disorders, Fifth Edition
eGFR: Estimated Glomerular Filtration Rate
EWGSOP2: European Working Group on Sarcopenia in Older People 2
IADL: Instrumental Activities of Daily Living
MCP: Middle Cerebellar Peduncle
MNA-SF: Mini Nutritional Assessment–Short Form
MRI: Magnetic Resonance Imaging
MRPI: Magnetic Resonance Parkinsonism Index
MSA: Multiple System Atrophy
OR: Odds Ratio
PD: Parkinson’s Disease
POMA: Performance-Oriented Mobility Assessment
PSP: Progressive Supranuclear Palsy
RAAS: Renin–Angiotensin–Aldosterone System
SCP: Superior Cerebellar Peduncle
SD: Standard Deviation
SGLT2: Sodium–Glucose Cotransporter-2
sMMSE: Standardized Mini-Mental State Examination
TSH: Thyroid-Stimulating Hormone
TUG: Timed Up and Go
YGDS: Yesavage Geriatric Depression Scale

## References

[1] Tinetti ME, Speechley M, Ginter SF. Risk factors for falls among elderly persons living in the community. N Engl J Med. 1988;319(26):1701–7. 10.1056/NEJM198812293192604 3205267

[2] Rubenstein LZ. Falls in older people: epidemiology, risk factors and strategies for prevention. Age Ageing. 2006;35(Suppl 2):ii37–41. 10.1093/ageing/afl084 16926202

[3] Yogev-Seligmann G, Hausdorff JM, Giladi N. The role of executive function and attention in gait. Mov disorders: official J Mov Disord. 2008;23(3):329–42. quiz 472. 10.1002/mds.21720 PMC2535903 18058946

[4] Montero-Odasso M, Verghese J, Beauchet O, Hausdorff JM. Gait and cognition: a complementary approach to understanding brain function and the risk of falling. J Am Geriatr Soc. 2012;60(11):2127–36. 10.1111/j.1532-5415.2012.04209.x PMC3498517 23110433

[5] Warmuth-Metz M, Naumann M, Csoti I, Solymosi L. Measurement of the midbrain diameter on routine magnetic resonance imaging: a simple and accurate method of differentiating between Parkinson disease and progressive supranuclear palsy. Arc Neurol. 2001;58(7):1076–9. 10.1001/archneur.58.7.1076 11448296

[6] Scheffler M, Maréchal B, Boto J, Lövblad KO, Vargas MI. A method for fast automated assessment of the magnetic resonance parkinsonism index. Neuroradiology. 2020;62(6):747–51. 10.1007/s00234-020-02380-5 32095870

[7] Quattrone A, Nicoletti G, Messina D, et al. MR imaging index for differentiation of progressive supranuclear palsy from Parkinson disease and the Parkinson variant of multiple system atrophy. Radiology. 2008;246:214–21. 10.1148/radiol.2453061703 17991785

[8] American Psychiatric Association. Diagnostic and statistical manual of mental disorders. 5th ed. Washington, DC: American Psychiatric Association; 2013.

[9] Durmaz B, Soysal P, Ellidokuz H, Isik AT. Validity and reliability of geriatric depression scale-15 (short form) in Turkish older adults. North clin Istanb. 2018;5(3):216–20. 10.14744/nci.2017.85047 PMC6323561 30688929

[10] Lawton MP, Brody EM. Assessment of older people: self-maintaining and instrumental activities of daily living. Gerontologist. 1969;9(3):179–86. 5349366

[11] Mahoney FI, Barthel D. Functıonal Evaluatıon: The Barthel Index. Md State Med J. 1965;14:61–5. 14258950

[12] Tinetti ME. Performance-oriented assessment of mobility problems in elderly patients. J Am Geriatr Soc. 1986;34(2):119–26. 10.1111/j.1532-5415.1986.tb05480.x 3944402

[13] Fried LP, Tangen CM, Walston J, et al. Frailty in older adults: evidence for a phenotype. J Gerontol Biol Sci Med Sci. 2001;56:M146–56. 10.1093/gerona/56.3.m146 11253156

[14] Cruz-Jentoft AJ, Bahat G, Bauer J, et al. Sarcopenia: revised European consensus on definition and diagnosis. Age Ageing. 2019;48:16–31. 10.1093/ageing/afy169 PMC6322506 30312372

[15] Rubenstein LZ, Harker JO, Salvà A, Guigoz Y, Vellas B. Screening for undernutrition in geriatric practice: developing the short-form mini-nutritional assessment (MNA-SF). J Gerontol Biol Sci Med Sci. 2001;56(6):M366–72. 10.1093/gerona/56.6.m366 11382797

[16] Molloy DW, Alemayehu E, Roberts R. Reliability of a Standardized Mini-Mental State Examination compared with the traditional Mini-Mental State Examination. Am J Psychiatry. 1991;148(1):102–5. 10.1176/ajp.148.1.102. 1984692

[17] Colón-Emeric CS, McDermott CL, Lee DS, Berry SD. Risk Assessment and Prevention of Falls in Older Community-Dwelling Adults: A Review. JAMA. 2024;331(16):1397–406. 10.1001/jama.2024.1416 PMC12224174 38536167

[18] Cheng MH, Chang SF. Frailty as a Risk Factor for Falls Among Community Dwelling People: Evidence From a Meta-Analysis. J Nurs Scholarsh: official publication Sigma Theta Tau Int Honor Soc Nurs. 2017;49(5):529–36. 10.1111/jnu.12322 28755453

[19] Chantanachai T, Sturnieks DL, Lord SR, Payne N, Webster L, Taylor ME. Risk factors for falls in older people with cognitive impairment living in the community: Systematic review and meta-analysis. Ageing Res Rev. 2021;71:101452. 10.1016/j.arr.2021.101452. 34450352

[20] Jacob L, Shin JI, Kostev K et al. Prospective association between multimorbidity and falls and its mediators. J Clin Med 2022;11(15):4470. 10.3390/jcm11154470. PMC9370027 35956086

[21] Center JR, Nguyen TV, Schneider D, et al. Mortality after all major types of osteoporotic fracture in men and women. Lancet. 1999;353:878–82. 10.1016/S0140-6736(98)09075-8 10093980

[22] Masnoon N, Shakib S, Kalisch-Ellett L, Caughey GE. What is polypharmacy? A systematic review of definitions. BMC Geriatr. 2017;17(1):230. 10.1186/s12877-017-0621-2. PMC5635569 29017448

[23] van der Velde N, Seppala LJ, Hartikainen S, et al. European position paper on polypharmacy and fall-risk-increasing drugs. Eur Geriatr Med. 2023;14:649–58. 10.1007/s41999-023-00824-8 PMC10447263 37452999

[24] Montero-Odasso MM, Kamkar N, Pieruccini-Faria F, et al. Evaluation of clinical practice guidelines on fall prevention. JAMA Netw Open. 2021;4:e2138911. 10.1001/jamanetworkopen.2021.38911 PMC8674747 34910151

[25] Fhon JR, Rodrigues RA, Neira WF, et al. Fall and its association with frailty syndrome in the elderly: systematic review with meta-analysis. Rev Esc Enferm USP. 2016;50:1005–13. 10.1590/S0080-623420160000700018 28198967

[26] Allen NE, Canning CG, Almeida LRS, et al. Interventions for preventing falls in Parkinson’s disease. Cochrane Database Syst Rev. 2022;6:CD011574. 10.1002/14651858.CD011574.pub2 PMC9169540 35665915

[27] Allen NE, Schwarzel AK, Canning CG. Recurrent falls in Parkinson’s disease: a systematic review. Parkinsons dis. 2013;2013:906274. 10.1155/2013/906274 PMC3606768 23533953

[28] Postuma RB, Berg D, Stern M, et al. MDS clinical diagnostic criteria for Parkinson’s disease. Mov disorders: official J Mov Disorder Soc. 2015;30(12):1591–601. 10.1002/mds.26424 26474316

[29] Boxer AL, Yu JT, Golbe LI, Litvan I, Lang AE, Höglinger GU. Advances in progressive supranuclear palsy: new diagnostic criteria, biomarkers, and therapeutic approaches. Lancet Neurol. 2017;16(7):552–63. 10.1016/S1474-4422(17)30157-6 PMC5802400 28653647

[30] Quattrone A, Morelli M, Nigro S, et al. A new MR imaging index for differentiation of progressive supranuclear palsy-parkinsonism from Parkinson’s disease. Parkinsonism Relat disord. 2018;54:3–8. 10.1016/j.parkreldis.2018.07.016 30068492

[31] Quattrone A, Antonini A, Vaillancourt DE, et al. A New MRI Measure to Early Differentiate Progressive Supranuclear Palsy From De Novo Parkinson’s Disease in Clinical Practice: An International Study. Mov Dis: official J Mov Disorder Soc. 2021;36(3):681–9. 10.1002/mds.28364 PMC8330364 33151015

[32] Hussl A, Mahlknecht P, Scherfler C, et al. Diagnostic accuracy of MRPI and midbrain-to-pons ratio. Mov Disord. 2010;25:2444–51. 10.1002/mds.23351 20878992

